# Supplementary material for: Inferring urban polycentricity from the variability in human mobility patterns
Source: Sci Rep. 2023 Apr 7;13:5751. doi: 10.1038/s41598-023-33003-7 (PMC10082211; doi:10.1038/s41598-023-33003-7)
Supplement: Supplementary file 1 — Supplementary Information. [file 41598_2023_33003_MOESM1_ESM.pdf]

Supplementary Information

1 Sensitivity analysis

In order to counter the possible arbitrariness in our choice of nucleus, we provide here a sensitivity analysis. The sensitivity analysis is performed by considering as nucleus all the stations in the transport system corresponding to each city which are within a 3 km radius from the initial choice of nucleus, i.e. Piccadilly Circus for London and City Hall for Seoul. Four separate supplementary files in .csv format are provided corresponding to Lonon and Seoul, for the two-component Poisson mixture model ('Supplementary Table S1' and 'Supplementary Table S2') and the three-component Poisson mixture model ('Supplementary Table S3' and 'Supplementary Table S4'). In each of these tables, the rows represent the different choices of nucleus. The columns are the estimated parameters (and their standard error) that define the regression line describing the relation between the mean of a component of the mixture model and the network distance between the chosen nucleus and a given destination station. The columns also include the correlation coefficient R and its p-value, as well as other fields discussed in the section titled 'Model selection'.

In order to quantify the variability of the estimated parameters that characterise the regression line under different choices of nucleus, we compute the relative standard deviation of each parameter (standard deviation divided by average, expressed as a percentage). As mentioned above, we consider as nucleus all the stations within a distance of 3 km from the main nucleus. The relative standard deviation for each parameter and city are gathered in Table 1 and Table 2 for the two-component and three-component Poisson mixture models respectively.

Table 1. Relative standard deviation of regression parameters obtained for the two-component Poisson mixture model under different choices of nucleus within a 3km radius of the main nucleus, for London and Seoul.

		London	Seoul
**Intercept**	Proximal	5.55%	0.49%
	Distal	4.17%	2.40%
**Slope**	Proximal	22.15%	9.10%
	Distal	12.15%	3.97%

Table 2. Relative standard deviation of regression parameters obtained for the three-component Poisson mixture models under different choices of nucleus within a 3km radius of the main nucleus, for London and Seoul.

		London	Seoul
**Intercept**	Proximal	5.58%	1.32%
	Medial	6.35%	2.05%
	Distal	6.15%	2.57%
**Slope**	Proximal	39.10%	118.94%
	Medial	8.15%	9.73%
	Distal	16.58%	7.96%

2 Weights of Poisson mixture model components

Figure 1 shows the relationship between d_{1i} and the weights corresponding to each component of the Poisson mixture model for station S_i. For the two-component mixture, we focus on the behaviour of the proximal weight w_i^p only, since $w_i^d = 1 - w_i^p$ and for the three-component mixture, we focus on the proximal and distal weights w_i^p and w_i^d, since $w_i^m = 1 - (w_i^p + w_i^d)$. From Figure 1, we conclude that there is no significant linear correlation between d_{1i} and the weight corresponding to the different components. This also suggests that, as d_{1i} increases, there is no obvious change in the passengers' preference to travel at local or city-wide scale.

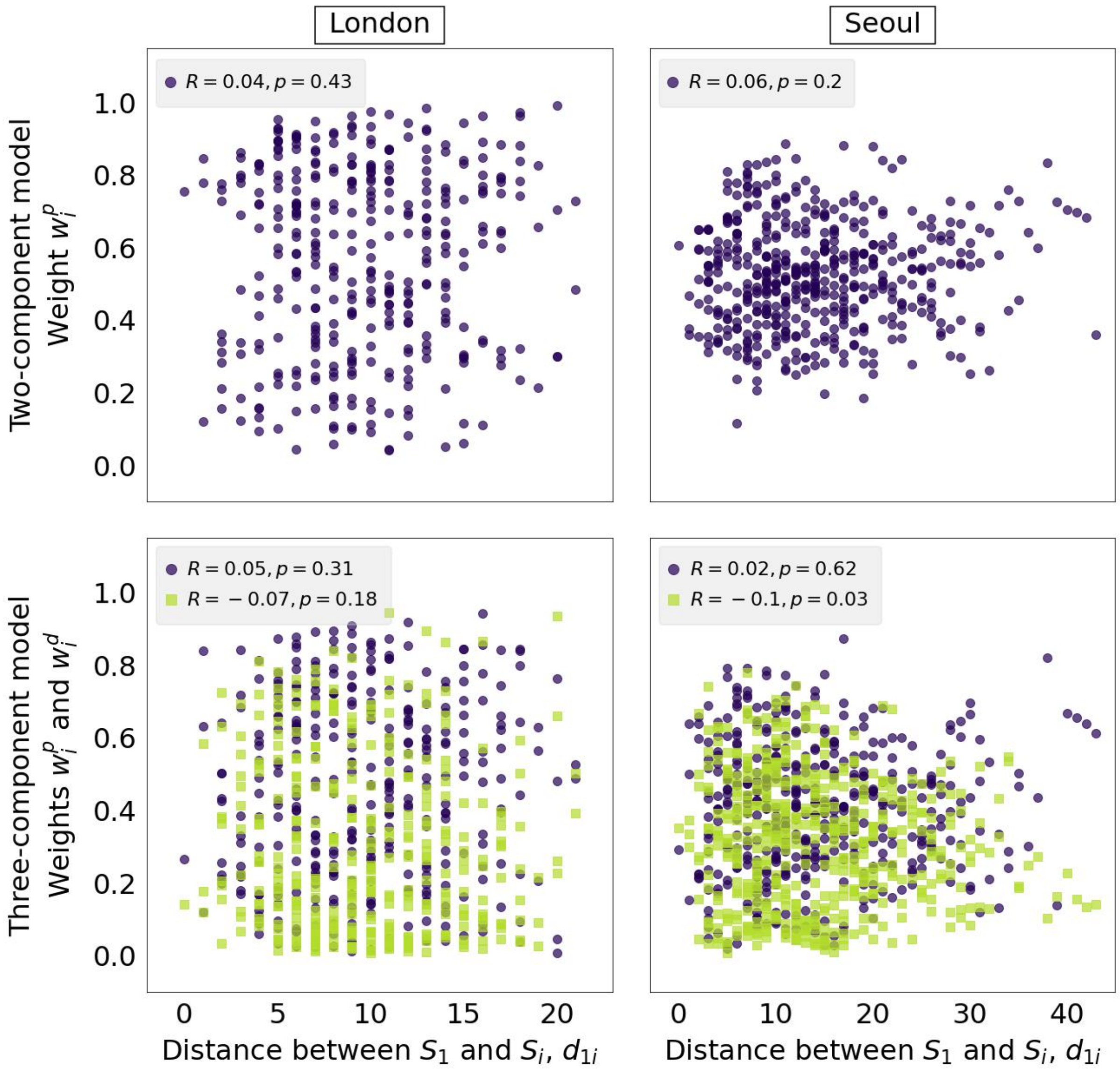

Figure 1. Relationship between td_{1i} and the weights corresponding to each component of the Poisson mixture model for station S_i

3 Model selection

We use the Bayesian Information Criterion (BIC) as a criterion for model selection. Our modelling approach has a multilevel structure, where the first level aims to describe the distribution of lengths of journeys terminating at each station, and the second level aims to model the relation between the mean length of journeys terminating at each station and the distance from the nucleus to that station. We apply the BIC only to the first level, in order to compare the suitability of two-component and a three-component Poisson mixture models. For each city and for each model, we obtain the values of BIC showed in Table 3. The values showed in Table 3 correspond to the main nucleus in each city, i.e. Piccadilly Circus and City Hall. Values of BIC for other choices of nucleus can be found in the Supplementary Tables S1-S4, where each row corresponds to a choice of nucleus and the columns included different terms for the computation of BIC: 'loglikelihood' (the log-likelihood), 'no_parameters' (the number of parameters), 'dof' (the number of degrees of freedom which is equal to the number of data points in the model) and 'BIC' (the Bayesian Information Criterion).

The values of BIC are generally high due to the large number of data points in each city. However, the BIC is consistently lower for the three-component Poisson mixture model, suggesting that this model should be selected. The fact that the BIC is lower in this instance suggests that adding a third component to the mixture results in a better description of the data despite the increase in the penalty term due to the larger number of parameters to be estimated. Specifically, for N stations, the

two-component Poisson mixture model is characterised by $4 \times N$ parameters (μ_i^p, μ_i^d, w_i^p and w_i^d for $i = 1,...,N$) and the three-component Poisson mixture model is characterised by $6 \times N$ parameters (μ_i^p, μ_i^m, μ_i^d, w_i^p, w_i^m and w_i^d for $i = 1,...,N$). In addition, the Supplementary Tables S1-S4 show that choosing Piccadilly Circus and City Hall as the nucleus for London and Seoul respectively also yields the lowest BIC, both for the two-component and the three-component Poisson mixture models.

Table 3. Bayesian Information Criterion corresponding to the Poisson mixture models with $K = 2, 3$ components for London and Seoul.

	$K = 2$	$K = 3$
**London**	17642212.73	17493588.44
**Seoul**	37636956.46	36820261.3
